# Semipermeable Mixed Phospholipid-Fatty Acid Membranes Exhibit K^+^/Na^+^ Selectivity in the Absence of Proteins

**DOI:** 10.3390/life10040039

**Published:** 2020-04-14

**Authors:** Xianfeng Zhou, Punam Dalai, Nita Sahai

**Affiliations:** 1 Department of Polymer Science, University of Akron, Akron, OH 44325, USA; xianfeng@uakron.edu (X.Z.); pdalai@uakron.edu (P.D.); 2 Key Lab of Biobased Polymer Materials of Shandong Provincial Education Department, College of Polymer Science and Engineering, Qingdao University of Science and Technology, Qingdao 266042, China

**Keywords:** membrane, prebiotic chemistry, K^+^/Na^+^ gradient, energy, origin of life

## Abstract

Two important ions, K^+^ and Na^+^, are unequally distributed across the contemporary phospholipid-based cell membrane because modern cells evolved a series of sophisticated protein channels and pumps to maintain ion gradients. The earliest life-like entities or protocells did not possess either ion-tight membranes or ion pumps, which would result in the equilibration of the intra-protocellular K^+^/Na^+^ ratio with that in the external environment. Here, we show that the most primitive protocell membranes composed of fatty acids, that were initially leaky, would eventually become less ion permeable as their membranes evolved towards having increasing phospholipid contents. Furthermore, these mixed fatty acid-phospholipid membranes selectively retain K^+^ but allow the passage of Na^+^ out of the cell. The K^+^/Na^+^ selectivity of these mixed fatty acid-phospholipid semipermeable membranes suggests that protocells at intermediate stages of evolution could have acquired electrochemical K^+^/Na^+^ ion gradients in the absence of any macromolecular transport machinery or pumps, thus potentially facilitating rudimentary protometabolism.

## 1. Introduction

K^+^ is the dominant ion in virtually all living cells and every mammalian cell has a high intra-cellular K^+^ content. Na^+^ is the most prominent cation in extra-cellular fluid. The unequal distribution of K^+^/Na^+^ and their selective movement through the membrane are primarily responsible for generating the resting membrane potential. In modern cells, this distribution of K^+^/Na^+^ is mediated by K^+^/Na^+^ channels and K^+^/Na^+^-ATPase, an active transporter pumping ions against the gradient [1,2,3]. This raises an interesting evolutionary question: did the high K^+^/Na^+^ ratio in the cytoplasm evolved actively for functional reasons or was it a result of passive processes? According to the “chemistry conservation principle” [4], the composition of the cellular cytosol is more conservative than that of the changing external environment and could be a physiological fossil of early metabolism to retain information of ancient environmental conditions. Based on this principle, it was previously suggested that the high K^+^/Na^+^ ratio of modern cells is a remnant of the first cells that evolved on Earth [5]. Taking high K^+^/Na^+^ ratio as a key search criterion, it was suggested that the most suitable hatcheries for the protocells were at inland geothermal fields with ponds of condensed and cooled geothermal vapor having high K^+^/Na^+^ ratios [5]. However, using today’s cells to mirror primordial protocells or ancient evolutionary habitats is questionable, because protocell membranes that were initially leaky could have gradually evolved to be less permeable, and therefore, any modern environment that matches this intra-cellular composition could be purely coincidental. Despite the discussion above, it is difficult to conclude that the high K^+^/Na^+^ ratio is independent of the nature of the early environment, because the transport of K^+^/Na^+^ across the cell membrane is essential to many cell functions and was likely present even in protocells [6].

The bottom-up assembly of a protocell, defined here as a membrane-bound prebiotic entity capable of both self-replication and metabolism, has been a grand challenge in the Origins of Life (OoL) field. It has been stated eloquently that “one cannot speak of a protocell type, but rather a lineage of protocell systems that slowly evolved from simple self-assembled molecular systems towards pre-cellular entities capable of self-sustenance and self-replication” [7]. In the present study, the term “protocells” will be used to refer to simple lipid vesicles, intermediate evolutionary states, and more complex pre-cellular entities. The most primitive cell membranes were likely formed from single-chain amphiphiles (SCAs), such as fatty acids and their derivatives, that were present in the prebiotic environment [8,9,10,11,12,13,14,15]. Membranes composed of short chain SCAs are leaky (reviewed in 15). The presence of high membrane permeability in the protocells would mean that they could have prevented the potential damage induced by osmotic pressure but could not have retained and accumulated K^+^ over Na^+^ and the small metabolites generated by primitive metabolism in their interior. In the presence of phospholipids synthesized abiotically [16,17,18,19,20], mixed lipid membranes would have formed in protocells at intermediate stages of evolution. The evolution of SCA lipid membranes to mixed fatty acid-phospholipid and eventually to phospholipid-enriched membranes could have been driven by environmental pressures, such as the presence of Mg^2+^ ions in the environment [14,21]. It has been shown that divalent cations can selectively bind to fatty acids and abstract them from mixed fatty acid-phospholipid membranes [14]. With increasing phospholipid content, the protocells would have eventually become impermeable to ions. In the absence of complex protein-based ion transport machinery across the membrane, the increasing presence of phospholipid in the membrane could have been fatal for protocells, because there would have been no way to maintain osmotic and electrical equilibria across the membranes. This could have served as an evolutionary pressure to evolve ion-transport membrane proteins [22]. To address the issue of protocell membrane permeability and stability from the most primitive to intermediate- and later-stage membrane compositions, we were motivated to examine K^+^ and Na^+^ permeability across fatty acid, mixed fatty acid-phospholipid, and phospholipid membranes. 

## 2. Materials and Methods 

### 2.1. Materials

**Figure 1:**
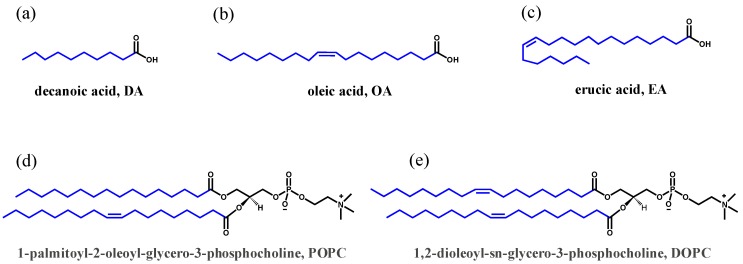
Structures of the various fatty acids and phospholipids used in the present study: (**a**) decanoic acid, DA; (**b**) oleic acid, OA; (**c**) erucic acid, EA; (**d**) 1-palmitoyl-2-oleoyl-glycero-3-phosphocholine, POPC; and (**e**) 1,2-dioleoyl-sn-glycerol-3-phosphocholine, DOPC.

Phospholipids (1,2-diheptanoyl-*sn*-glycero-3-phosphocholine (C7:0, DHPC), 1,2-Dioleoyl-*sn*-glycero-3-phosphocholine (C18:1, DOPC), and 1-palmitoyl-2-oleoyl-*sn*-glycero-3-phosphocholine (C16:0-C18:1, POPC) and single-chain fatty acids (oleic acid (C18:1, OA) and erucic acid (C22:1, EA)) were obtained from Avanti Polar Lipids^®^ (Figure 1). Potassium-binding benzofuran isophthalate (PBFI) tetra-ammonium salt and sodium-binding benzofuran isophthalate (SBFI) tetra-ammonium salts were obtained from Invitrogen (Thermo Fisher Scientific, Waltham, MA, USA). Valinomycin, monensin, pyranine, and calcein were obtained from Sigma-Aldrich (St. Louis, MO, USA). Unless otherwise specified, all other chemicals were purchased from Sigma-Aldrich at the highest available purity and used without further purification. All solutions were prepared with ultrapure deionized water with a resistivity of 18.2 MΩ.cm (Barnstead™ GenPure™ xCAD Plus, Thermo Scientific, Rockford, IL, USA). 

### 2.2. Vesicle Preparation and Characterization

Fatty acids, phospholipids, and mixed lipids were dissolved in chloroform. The lipids were dried using a stream of nitrogen gas for over 3 h to yield a thin film on the walls of glass tubes. Lipid films were subsequently rehydrated by vigorous vortexing and sonicated with a solution of dyes (calcein (20 mM), PBFI/pyranine (100/50 µM), or SBFI/pyranine (100/50 µM)) in 50 mM bicine buffer (pH 8.5). The pH of the buffer was adjusted with trimethylamine (TEA) to pH 8.5. Afterwards, samples were briefly sonicated and vortexed. After brief sonication, the samples were freeze-thawed five times to ensure greater encapsulation of the dyes. Thereafter, the vesicle suspensions were gently agitated by end-over-end rotation overnight prior to use, in order to ensure that the vesicles remained stable. The suspension of vesicles was allowed to tumble overnight and then subjected to low pressure-size exclusion chromatography (in a 10 cm × 1 cm column) to separate the dye-encapsulating vesicles from the un-encapsulated free dye. The column was filled with Sephadex G-50 medium beads (Sigma Aldrich, St. Louis, MO, USA)). The mobile phase was a 50 mM bicine/TEA buffer at pH 8.5. Fractions were collected using a fraction collector (FC204, Gilson) in a 96-well plate. Fluorescence was measured in a microplate reader (Synergy H1, BioTek Instruments, Winooski, VT, USA) and the fractions containing vesicles were pooled (Figure S1). Vesicle size was measured by dynamic light scattering (Zetasizer Nano ZS, Malvern). Images of the vesicles were taken on a IX51 Epifluorescence Microscope (Olympus Co., Japan) equipped with a fluorescent light source and filters. All vesicles were used between 4 and 24 h after purification.

### 2.3. Calcein Leakage Assay

The stabilities of vesicles of different compositions were assessed by quantifying the leakage of calcein [23]. The kinetics of the release of calcein were monitored for 24 h (at an excitation/emission wavelength of 495/530 nm). 

The percentage of encapsulation was calculated according to the following Equation (1):

(1) $$\mathrm{Encapsulation}\;\left(\%\right)=100\times \left(1-\frac{F_{t}-F_{0}}{F_{f}-F_{0}}\right)$$

where F_t_ is the fluorescence at time t, F_0_ is the fluorescence at time zero, and F_f_ is the fluorescence after the addition of Triton X-100. The leakage (%) is the 100-encapsulation %.

### 2.4. K^+^ and Na^+^ Calibration and Measurements

The PBFI molecule is a crown ether compound, linked via its nitrogen atoms to K^+^ and to benzofuran fluorophore-bearing isophthalate groups as additional ligating centers. For calibration, the excitation spectra of PBFI (1 µM) at different K^+^ concentrations were first obtained. 

According to the law of mass action, PBFI binds to K^+^ according to:

(2) $$K_{d,\mathrm{app}}=\left(\left[\mathrm{PBFI}\right]\times \left[K^{+}\right]\right)/\left[\mathrm{KPBFI}\right]$$

where K_d,app_ is the apparent dissociation constant. 

Since the fluorescence of the KPBFI complex is higher than that of free PBFI, fluorescence increases as the concentration of KPBFI increases (Figure S2), according to Equation (3):

(3) $$F_{K}=F_{0}+\gamma \times \left[\mathrm{KPBFI}\right]$$

where F_0_ and F_k_ are, respectively, the fluorescence of the dye in the absence or presence of K^+^, and γ is a constant. 

Therefore, the binding isotherm, in terms of fluorescence, is:

(4) $$f=\left(F_{k}-F_{0}\right)/\left(F_{\max}-F_{0}\right)=\left[K^{+}\right]/\left(K_{d,\mathrm{app}}+\left[K^{+}\right]\right)$$

where F_max_ is the fluorescence of PBFI in the presence of a saturating K^+^ concentration. We define parameter f as ($F_{k}-F_{0}$)/$\left(F_{\max}-F_{0}\right)$, and using the Scatchard plot, the slope should be (−1/K_d,app_).

Using the bicine/TEA buffer at different pH values (pH 6.75–9), we observed that the values of K_d,app,K_^+^ (4.01 ± 0.29 mM) and K_d,app,Na_^+^ (5.99 ± 0.51 mM) remained almost unaffected when the pH was lower than 8.5 but higher than 6.75 (Figure S3 and Table S1). 

Since the same K_d,app_ values were determined for free PBFI in buffer as for encapsulated PBFI in vesicles [24], we could use Equation (5) to calculate the concentration of intra-vesicular K^+^ according to:

(5) $$\left[K^{+}\right]=K_{d,\mathrm{app}}/\left(\left(F_{m}-F_{k}\right)/\left(F_{k}-F_{0}\right)\right)$$

Based on Fick’s law of diffusion, the uptake rate of K^+^ (ds/dt) through the membrane into a vesicle (volume V) can be represented as:

(6) $$\frac{\mathrm{ds}}{\mathrm{dt}}=P\times A\times \Delta C$$

and

(7) $${\mathrm{dC}}_{\mathrm{in}}=\frac{\mathrm{ds}}{V}$$

where P is the permeability coefficient, A is the surface area of vesicle, and ΔC is the difference in K^+^ concentration between the interior (C_in_) and exterior (C_ex_) compartments (C_ex_ − C_in_).

The two equations can be simplified as:

(8) $$\frac{{\mathrm{dC}}_{\mathrm{in}}}{\mathrm{dt}}=\frac{\mathrm{PA}}{V}\left(C_{\mathrm{ex}}-{\;C}_{\mathrm{in}}\right)$$

After integration, the concentration of K^+^ in vesicle C_in(t)_ after time t can be expressed as:

(9) $$\frac{C_{\mathrm{ex}}-C_{\mathrm{in}\left(t\right)}}{C_{\mathrm{ex}}-C_{0}}=\exp\left[\mathrm{PAt}/V\right]$$

where C_0_ is the concentration of K^+^ in vesicle at t = 0 and C_ex_ is constant because of the large exterior volume.

Therefore, the permeability coefficient can be calculated according to Equation (10):

(10) $$P=\frac{V}{\mathrm{At}}\ln\left(1-\frac{C_{\mathrm{in}\left(t\right)}}{C_{\mathrm{ex}}}\right)=\frac{r}{3t}\ln\left(1-\frac{C_{\mathrm{in}\left(t\right)}}{C_{\mathrm{ex}}}\right)$$

where V, A, and r, respectively, are the volume, area, and radius of the vesicle. This procedure is also used to calculate the Na^+^ permeability coefficient using SBFI.

### 2.5. K^+^ and Na^+^ Permeability Experiments

PBFI and SBFI are molecules that fluoresce upon binding K^+^ and Na^+^, respectively (Figure S2). Valinomycin and monensin are K^+^ and Na^+^-selective molecules, respectively, that can bind and transport these ions across the membrane. Vesicles were prepared with encapsulated PBFI/pyranine or SBFI/pyranine. Twenty millimolar KCl or NaCl and a specific ion transporter (valinomycin or monensin) were added to the exterior of the vesicles, and the fluorescence intensity change of PBFI or SBFI (Excitation/Emission = ~336/506 nm) inside the vesicle was measured as a function of time, for up to 100 min. Valinomycin and monensin were used, respectively, as positive controls for K^+^ and Na^+^ ion permeability.

## 3. Results

### 3.1. Calcein Leakage Assay

We first examined the stability of various SCAs and phospholipid membranes using the calcein leakage assay. Since the permeation rates and translocation mechanisms of ions through membranes are functions of membrane thickness [6], we first used a short-chain fatty acid (C10, DA, decanoic acid) and its corresponding alcohol (DOH, decanol) to form vesicles in a 2:1 ratio (Figure S3). This prebiotically plausible membrane could feature high intrinsic ion permeability, allowing non-facilitated membrane transport [25]. However, we found that the DA/DOH vesicles were not stable and started to allow the the fluorescent dyes to leak out, even during the preparation and separation in 50 mM bicine buffer (pH 8.5). The SCAs with longer alkyl chains, such as OA (C18:1) and EA (C22:1), as well as phospholipids such as DOPC (C18:1) and POPC (C16:0-C18:1), were also tested for their stability. The calcein leakage assay results show that vesicles formed with longer single-chain fatty acids or phospholipids are fairly stable over 24 h, retaining >97% of the encapsulated fluorescent calcein dye (Figure S3). 

### 3.2. K^+^ Permeability of Pure Fatty Acid and Pure Phospholipid Membranes

**Figure 2:**
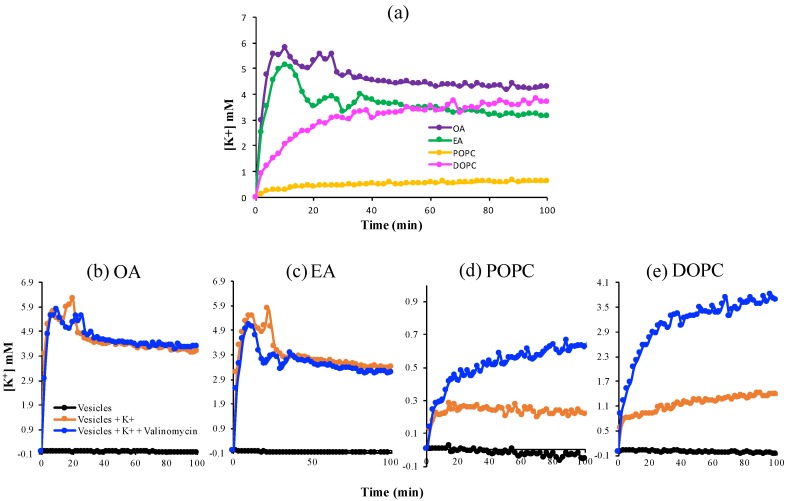
The permeability of K^+^ across various lipid membranes. (**a**) A comparison of fatty acid versus phospholipid membranes; (**b**–**e**) Permeability in the absence and presence of valinomycin, a K^+^-ion transporter, across membranes, as a positive control.

Next, we investigated the dynamics of K^+^ passage across these more stable OA, EA, DOPC, and POPC membranes (Figure 2). Valinomycin, a natural, lipid-soluble peptide that selectively binds K^+^ and facilitates its transfer across the lipid membrane, was added into the vesicles as a positive control for K^+^-permeability. After addition of 20 mM KCl to the exterior of the vesicles, the single-chain fatty-acid vesicles showed a peak at about 10–20 min followed by a decrease and quasi-steady state, in the presence and absence of valinomycin, which implies that these vesicles are permeable to K^+^. In contrast, the K^+^ concentration increased smoothly with time and reached a steady-state plateau for the phospholipid systems and permeability was much lower in the absence of valinomycin. The fluctuations in the fatty acid system reflect the greater leakiness and instability of the fatty acid membranes compared to the phospholipid membranes. 

The kinetic data permitted us to use Fick’s Law to approximate the permeability coefficients of these membranes with respect to K^+^ (Equation (10)). In the absence of valinomycin, the measured apparent permeability coefficients for phospholipid vesicles were ~10^−13^ (m/s), close to values reported previously [26], which means that these membranes were impermeable to K^+^. The single-chain fatty-acid vesicles exhibited a 10-fold higher permeability to K^+^ as compared to the phospholipid vesicles. 

### 3.3. K^+^ and Na^+^ Permeability of Mixed Fatty Acid-Phospholipid Membranes

**Figure 3:**
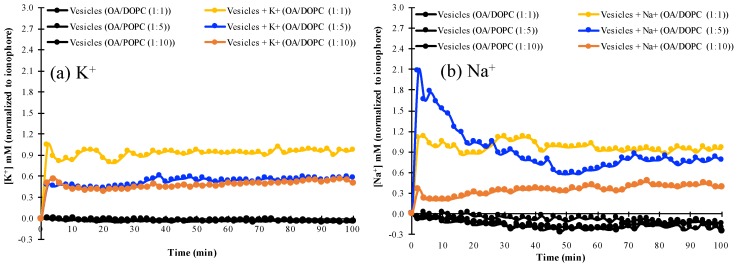
The permeability to (**a**) K^+^ and (**b**) Na^+^ of various mixed OA/DOPC membranes in the absence of ionophores, normalized to the respective concentrations in the presence of ionophores. The ionophore for K^+^ is valinomycin and the ionophore for Na^+^ is monensin.

We examined the permeability of mixed fatty acid/phospholipid vesicles with increasing phospholipid concentration to K^+^ and Na^+^, by monitoring the changes in intravesicular ion concentrations as ions permeated from the extravesicular solution into the vesicles. It was found that OA/DOPC (1:1) vesicles are permeable to K^+^, as shown by the similar fluorescence profile of the K^+^-specific PBFI indicator with or without valinomycin (Figure 3 and Figure S4). Similarly, this OA/DOPC (1:1) composition is also permeable to Na^+^ in the presence or absence of the Na^+^-specific ionophore, monensin (Figure 3 and Figure S5). By contrast, OA/DOPC (1:10) membranes are not permeable to either K^+^ or Na^+^. The decreased permeability of vesicle membranes could be explained by the intrinsically lower fluidity of phospholipids compared to single-chain fatty-acids [27]. However, OA/DOPC (1:5) vesicles discriminate towards Na^+^ over K^+^, and are more permeable to Na^+^ but relatively less permeable to K^+^ (Figure 3, Figures S4 and S5). 

These results are also seen in Table 1, where the concentrations of Na^+^ and K^+^ normalized to those obtained in the presence of their respective ionophores are almost equal for the OA/DOPC (1:1) system, whereas the values for Na^+^ are smaller than those for K^+^ in the OA/DOPC (1:10 system). However, the ionophore-normalized concentration of Na^+^ is greater than the corresponding value for K^+^ in the OA/DOPC (1:5) system, indicating a greater permeation of Na^+^ than of K^+^ across the membrane. The differences in the concentrations of K^+^ and Na^+^ (Table 1) also show that the OA/DOPC (1:5) vesicles exhibit a 10-fold higher permeability to Na^+^ than to K^+^. 

We also investigated the effect of K^+^ and Na^+^ on the stability of the vesicles to determine if osmostic pressure affected the permeability measurements by influencing membrane structure. The vesicle stability was estimated by determining the size of vesicles at different salt concentrations (Table S2). No differences were found in the sizes of the vesicles up to 75 mM NaCl or KCl, indicating no osmotic pressure effects. The vesicles remained intact and did not rupture or change in structure due to the presence of the salts. Therefore, the difference in ion permeability kinetics was mainly due to intrinsic differences in the membrane between the various mixed lipid compositions. 

## 4. Discussion

The data above show that pure fatty acid membranes and fatty acid-phospholipid membranes at relatively low phospholipid ratios (1:1) are permeable to both Na^+^ and K^+^. At relatively high ratios (1:10) of phospholipid content, the membranes are relatively impermeable to both ions. At a specific intermediate value of fatty acid to phospholipid (1:5), the membranes show greater permeability to Na^+^ than to K^+^. Here, we attempt to address the mechanism behind these observations. At low phospholipid content in the mixed lipid system, the properties of the membrane are dominated by the behavior of the fatty acids, which are known to form highly permeable membranes because of their greater fluidity. The fatty acid head group can bind cations and transport them across the membrane by the lipid molecule flip-flop mechanism or by local defects or transient pores in the membrane [28,29,30]. To address the selectivity for Na^+^ and K^+^ at an intermediate OA/DOPC ratio, the radii of hydrated and non-hydrated ions at the membrane interface have to be considered. The hydration shell of Na^+^ (0.45 nm) is larger than the hydration shell of K^+^ (0.3 nm) [31]. In addition, the Na^+^ hydration shell, formed by three water molecules, is quite stable, while the K^+^ ion cannot attract the water molecules and can only perturb the water structure in its immediate neighborhood [32]. Therefore, the hydrated Na^+^ ion should be less permeable by simple size exclusion. To realize a greater permeability of Na^+^ over K^+^ at the intermediate OA/DOPC ratio of 1:5, we speculate that OA must be able to dehydrate the cations [33,34]. The smaller size of the dehydrated Na^+^ ion compared to K^+^ then allows it to permeate through the membrane by the flip-flop mechanism, local defects, or transient pore mechanisms. Furthermore, Na^+^ has been shown to initiate the deprotonation of the carboxylic acids of fatty acids, even under neutral pH conditions [35]. The deprotonated headgroups could act to bind the ions. This energetically favorable binding interaction could balance the energy lost in the removal of the water molecules from the ion and would also be entropically favored because of the release of water molecules from the solvation sheath of the ion. At the OA/DOPC ratio of 1:10, there is an insufficient concentration of OA in the membrane to permit either ion to be dehydrated and transported across the membrane, while there is a sufficiently high phospholipid concentration to make the membrane relatively impermeable to both ions.

Several studies have proposed that the origin of life was at the surface of the Earth in “warm little ponds” such as geothermal pools [5,36,37,38]. In these environments, the salinity of the solutions is greater than the salinity of freshwater that may constitute neighboring surface run-off or neighboring streams or lakes. Most geochemical solutions have a higher Na^+^ than K^+^ content. When a protocell membrane first self-assembles from the environment, the intra-protocellular solution composition is the same as that of the environment. If a protocell self-assembled in a geothermal pool-like environment, then there would be an initially higher concentration of salts (compared to in the neighboring freshwater) in the intra-protocellular volume, with a K^+^/Na^+^ ratio <1. If the protocell was transported by surface runoff following a rainstorm to a freshwater stream or lake, the extra-protocellular volume would have a lower salinity than the intra-protocellular volume. The concentration gradient across the membrane would promote the permeation of both ions out of the vesicle across the membrane. For the population of protocells with fatty acid/phospholipid ratios in the intermediate range, Na^+^ ions would permeate more quickly than K^+^ ions, thus resulting in a net intra-protocellular K^+^/Na^+^ ratio >1. Thus, an electrochemical gradient would be established across the membrane that could be harvested for use in rudimentary protometabolism. As protocells evolved towards more phospholipid-enriched membrane compositions [14], the decreasing permeability would have acted as an environmental selection pressure to evolve ion channels and pumps.

## 5. Conclusions

We have shown that mixed fatty acid/phospholipid membranes of specific compositions show preferential permeability to Na^+^ over K^+^ without complex ion channels. The ion selectivity of specific mixed fatty acid/phospholipid membranes could have served as a mechanism for enabling weak protometabolism in protocells at intermediate stages of evolution in the absence of complex ion channels or pumps in the membrane.

## Figures and Tables

**Table 1:** The concentrations of Na^+^ or K^+^ cations normalized to those obtained in the presence of the ionophores inside mixed vesicles at specific time points.

Time (min)	OA/DOPC (1:1) (mM)		OA/DOPC (1:5) (mM)		OA/DOPC (1:10) (mM)	
	Na^+^	K^+^	Na^+^	K^+^	Na^+^	K^+^
2	1.1	1.05	2.07	0.48	0.36	0.5
10	1.05	0.83	1.52	0.48	0.21	0.42
24	0.95	0.8	0.97	0.46	0.29	0.42
60	0.92	0.93	0.65	0.53	0.39	0.5
100	1.02	0.97	0.78	0.58	0.39	0.5

Error bars are between 0.01–0.07. After 60 min, concentration of both Na^+^ and K^+^ have almost reached plateau (see Figure 3).

## Supplementary Materials

The following are available online at https://www.mdpi.com/2075-1729/10/4/39/s1, Figure S1: A representative column purification of a sample of PBFI-pyranine-loaded vesicles (OA/DOPC (1:5)); Figure S2. Fluorescence spectra showing the increase in the fluorescence intensity of PBFI and Pyranine with change in pH and binding of K^+^ with PBFI; Figure S3. Calcein leakage assay to determine stability (permeability) of membranes of various lipids; Figure S4. Permeability of K^+^ across mixed OA/DOPC membranes in the absence (a) and presence (e) of ionophore: comparison of various OA/DOPC compositions. (b)–(d) permeability in the absence and presence of valinomycin, a K^+^-ion transporter across membranes, as a positive control for OA/DOPC ratios of 1:1 (b); (1:5) (c) and (1:10) (d); Figure S5. Permeability of Na^+^ across mixed OA/DOPC membranes in the (a) absence and (e) presence of ionophore: comparison of various OA/DOPC compositions. (b)–(d) permeability in the absence and presence of monensin, a Na^+^-ion transporter across membranes, as a positive control for OA/DOPC ratios of 1:1 (b); (1:5) (c) and (1:10) (d); Table S1: The dissociation constant, *K_d_,* for calculating the concentration of intra-vesicular Na^+^ or K^+^; Table S2: Effect of Na^+^ and K^+^ on the size of vesicles to determine if osmotic pressure affects vesicle size by changing membrane structure.

## References

[1] Holmgren M., Wagg J., Bezanilla F., Rakowski R.F., De Weer P., Gadsby D.C. (2000). Three distinct and sequential steps in the release of sodium ions by the Na^+^/K^+^-ATPase. Nature.

[2] Cannon S.C. (2004). Paying the price at the pump: Dystonia from mutations in a Na^+^/K^+^-ATPase. Neuron.

[3] Morth J.P., Pedersen B.P., Buch-Pedersen M.J., Andersen J.P., Vilsen B., Palmgren M.G., Nissen P. (2011). A structural overview of the plasma membrane Na^+^,K^+^-ATPase and H^+^-ATPase ion pumps. Nat. Rev. Mol. Cell Biol..

[4] Macallum A.B. (1926). The paleochemistry of the body fluids and tissues. Physiol. Rev..

[5] Mulkidjanian A.Y., Bychkov A.Y., Dibrova D.V., Galperin M.Y., Koonin E.V. (2012). Origin of first cells at terrestrial, anoxic geothermal fields. Proc. Nat. Acad. Sci. USA.

[6] Pohorille A., Deamer D. (2009). Self-assembly and function of primitive cell membranes. Res. Microbiol..

[7] Kee T.P., Monnard P.-A. (2016). On the emergence of a proto-metabolism and the assembly of early protocells. Elements.

[8] Deamer D.W., Pashley R. (1989). Amphiphilic components of the Murchison carbonaceous chondrite: Surface properties and membrane formation. Orig. Life Evol. Biosph..

[9] Komiya M., Shimoyama A., Harada K. (1993). Examination of organic compounds from insoluble organic matter isolated from some Antarctic carbonaceous chondrites by heating experiments. Geochim. Cosmochim. Acta.

[10] McCollom T.M., Ritter G., Simoneit B.R. (1999). Lipid synthesis under hydrothermal conditions by Fischer-Tropsch-type reactions. Orig. Life Evol. Biosph..

[11] Rushdi A.I., Simoneit B.R. (2001). Lipid formation by aqueous Fischer-Tropsch-type synthesis over a temperature range of 100 to 400 C. Orig. Life Evol. Biosph..

[12] Foustoukos D.I., Seyfried W.E. (2004). Hydrocarbons in hydrothermal vent fluids: The role of chromium-bearing catalysts. Science.

[13] Dalai P., Kaddour H., Sahai N. (2016). Incubating life: Prebiotic sources of organics for the origin of life. Elements.

[14] Dalai P., Ustriyana P., Sahai N. (2018). Aqueous magnesium as an environmental selection pressure in the evolution of phospholipid membranes on early earth. Geochim. Cosmochim. Acta.

[15] Dalai P., Sahai N., Kolb V. (2018). Protocell emergence and evolution. Handbook of Astrobiology.

[16] Hargreaves W., Mulvihill S., Deamer D. (1977). Synthesis of phospholipids and membranes in prebiotic conditions. Nature.

[17] Rao M., Eichberg J., Oró J. (1982). Synthesis of phosphatidylcholine under possible primitive Earth conditions. J. Mol. Evol..

[18] Maheen G., Tian G., Wang Y., He C., Shi Z., Yuan H., Feng S. (2010). Resolving the enigma of prebiotic C-O-P bond formation: Prebiotic hydrothermal synthesis of important biological phosphate esters. Heteroat. Chem..

[19] Albertsen A.N., Duffy C., Sutherland J.D., Monnard P.-A. (2014). Self-assembly of phosphate amphiphiles in mixtures of prebiotically plausible surfactants. Astrobiology.

[20] Patel B.H., Percivalle C., Ritson D.J., Duffy C.D., Sutherland J.D. (2015). Common origins of RNA, protein and lipid precursors in a cyanosulfidic protometabolism. Nat. Chem..

[21] Jin L., Kamat N.P., Jena S., Szostak J.W. (2018). Fatty acid/phospholipid blended membranes: A potential intermediate state in protocellular evolution. Small.

[22] Budin I., Szostak J.W. (2011). Physical effects underlying the transition from primitive to modern cell membranes. Proc. Nat. Acad. Sci. USA.

[23] Kendall D.A., MacDonald R.C. (1982). A fluorescence assay to monitor vesicle fusion and lysis. J. Biol.Chem..

[24] Venema K., Gibrat R., Grouzis J.-P., Grignon C. (1993). Quantitative measurement of cationic fluxes, selectivity and membrane potential using liposomes multilabelled with fluorescent probes. Biochim. Biophys. Acta Biomembr..

[25] Mansy S.S., Schrum J.P., Krishnamurthy M., Tobe S., Treco D.A., Szostak J.W. (2008). Template-directed synthesis of a genetic polymer in a model protocell. Nature.

[26] Costa P.F., Emilio M.G., Fernandes P.L., Ferreira H.G., Ferreira K.G. (1989). Determination of ionic permeability coefficients of the plasma membrane of Xenopus laevis oocytes under voltage clamp. J. Physiol..

[27] Lande M.B., Donovan J.M., Zeidel M.L. (1995). The relationship between membrane fluidity and permeabilities to water, solutes, ammonia, and protons. J. Gen. Physiol..

[28] Paula S., Deamer D.W. (1999). Membrane permeability barriers to ionic and polar solutes. Curr. Topics. Memb..

[29] Paula S., Volkov A., Van Hoek A., Haines T., Deamer D.W. (1996). Permeation of protons, potassium ions, and small polar molecules through phospholipid bilayers as a function of membrane thickness. Biophys. J..

[30] Wilson M.A., Pohorille A. (1996). Mechanism of unassisted ion transport across membrane bilayers. J. Am. Chem. Soc..

[31] Mancinelli R., Botti A., Bruni F., Ricci M.A., Soper A.K. (2007). Hydration of sodium, potassium, and chloride ions in solution and the concept of structure maker/breaker. J. Phys. Chem. B.

[32] Degrève L., Vechi S.M., Junior C.Q. (1996). The hydration structure of the Na+ and K+ ions and the selectivity of their ionic channels. Biochim. Biophys. Acta-Bioenerg..

[33] Dudev T., Lim C. (2010). Factors governing the Na^+^ vs K^+^ selectivity in sodium ion channels. J. Am. Chem. Soc..

[34] Dudev T., Lim C. (2014). Ion selectivity strategies of sodium channel selectivity filters. Acc. Chem. Res..

[35] Tang C.Y., Allen H.C. (2009). Ionic Binding of Na^+^ versus K^+^ to the carboxylic acid headgroup of palmitic acid monolayers studied by vibrational sum frequency generation Spectroscopy. J. Phys. Chem. A.

[36] Damer B., Deamer D. (2015). Coupled phases and combinatorial selection in fluctuating hydrothermal pools: A scenario to guide experimental approaches to the origin of cellular life. Life.

[37] Sutherland J.D. (2016). The origin of life—Out of the blue. Angew. Chem..

[38] Deamer D.W. (1997). The first living systems: A bioenergetic perspective. Microbiol. Mol. Biol. Rev..

