# Supplementary material for: Semipermeable Mixed Phospholipid-Fatty Acid Membranes Exhibit K+/Na+ Selectivity in the Absence of Proteins
Source: Life (Basel). 2020 Apr 14;10(4):39. doi: 10.3390/life10040039 (PMC7235748; doi:10.3390/life10040039)
Supplement: Supplementary file 1 [file life-10-00039-s001.zip › life-751863-supplementary updated.pptx]

## Slide 1
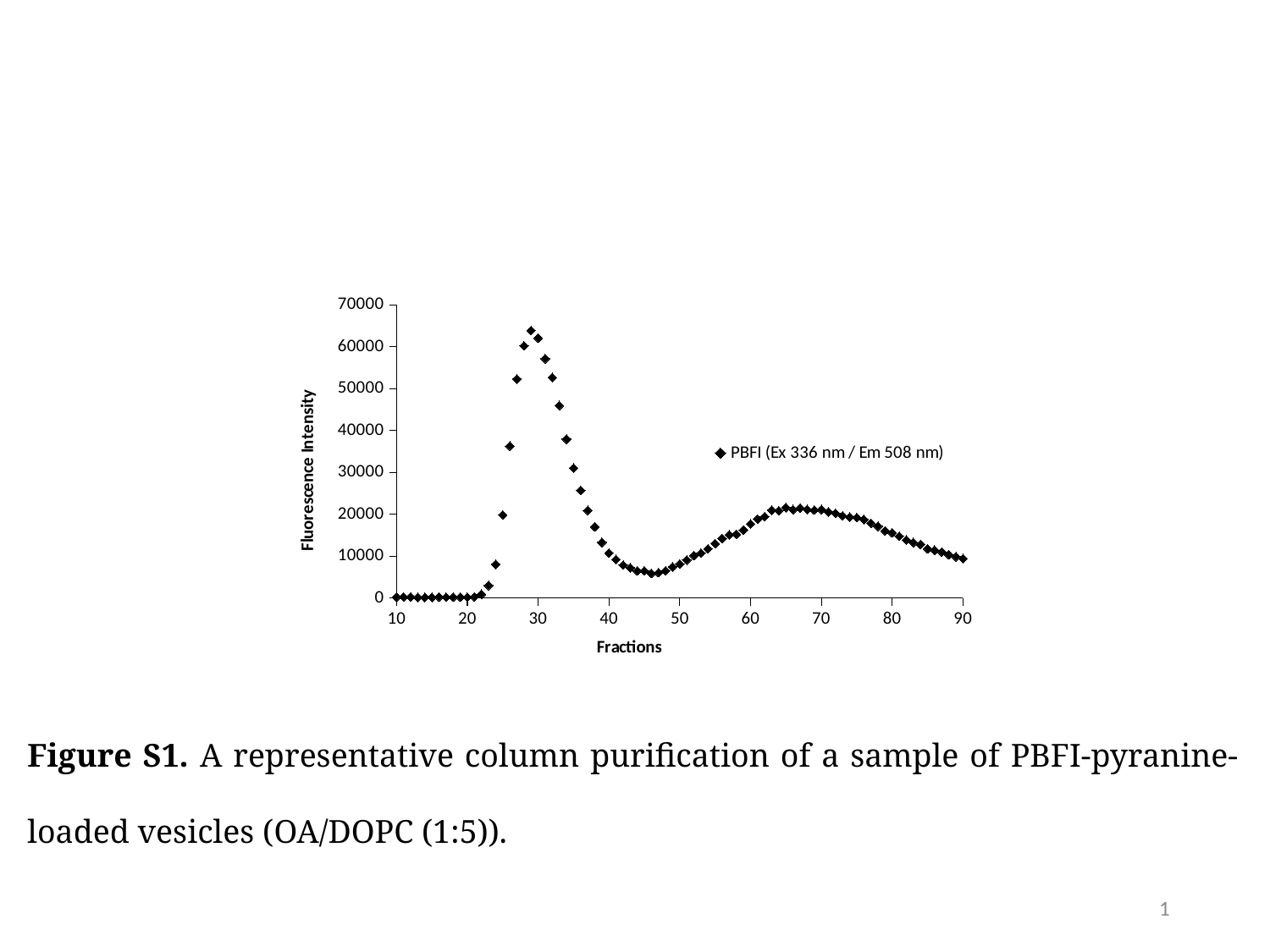

### Chart
| Category | |
|---|---|Figure S1. A representative column purification of a sample of PBFI-pyranine-loaded vesicles (OA/DOPC (1:5)).
1

## Slide 2
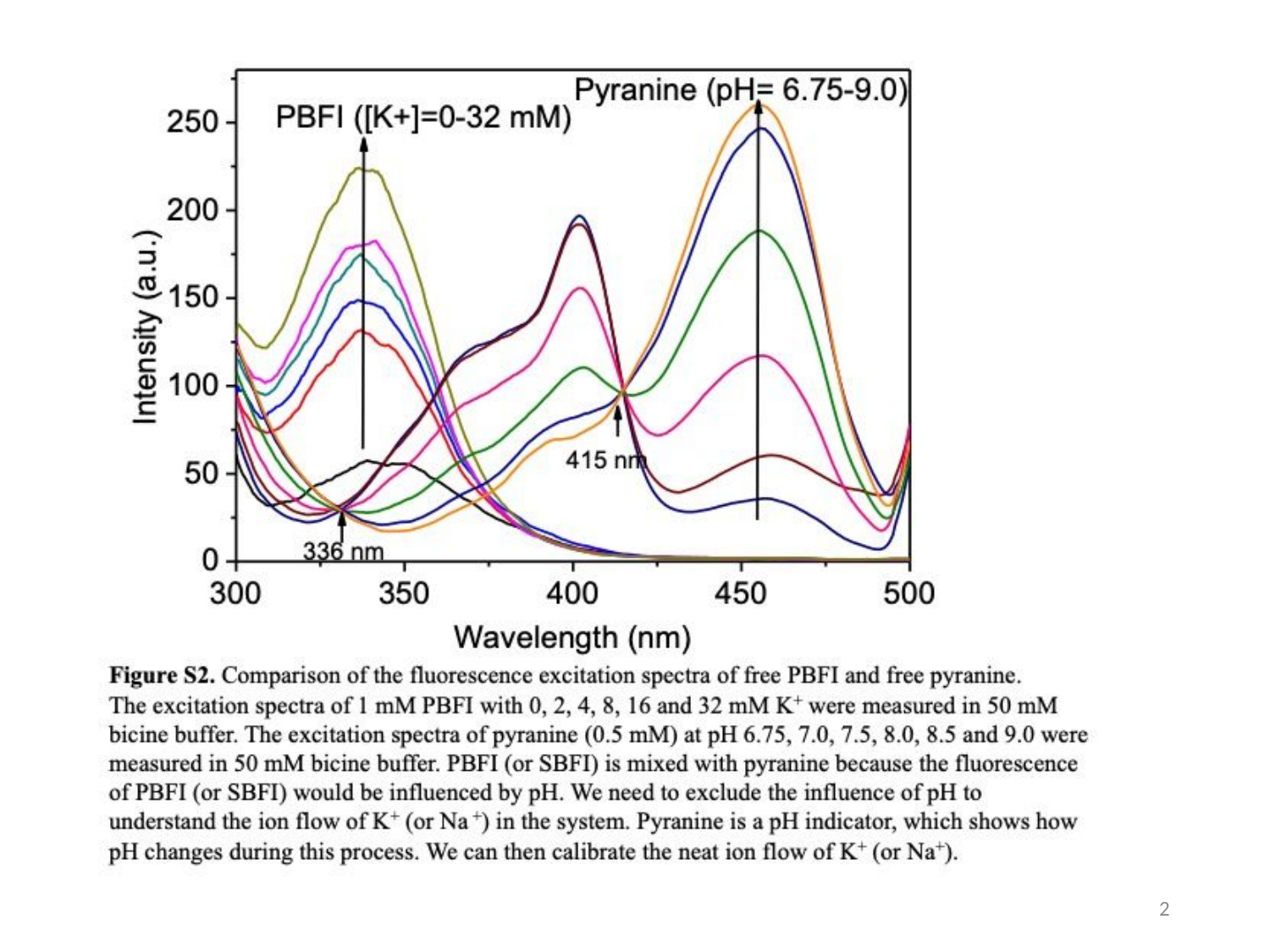

2

## Slide 3
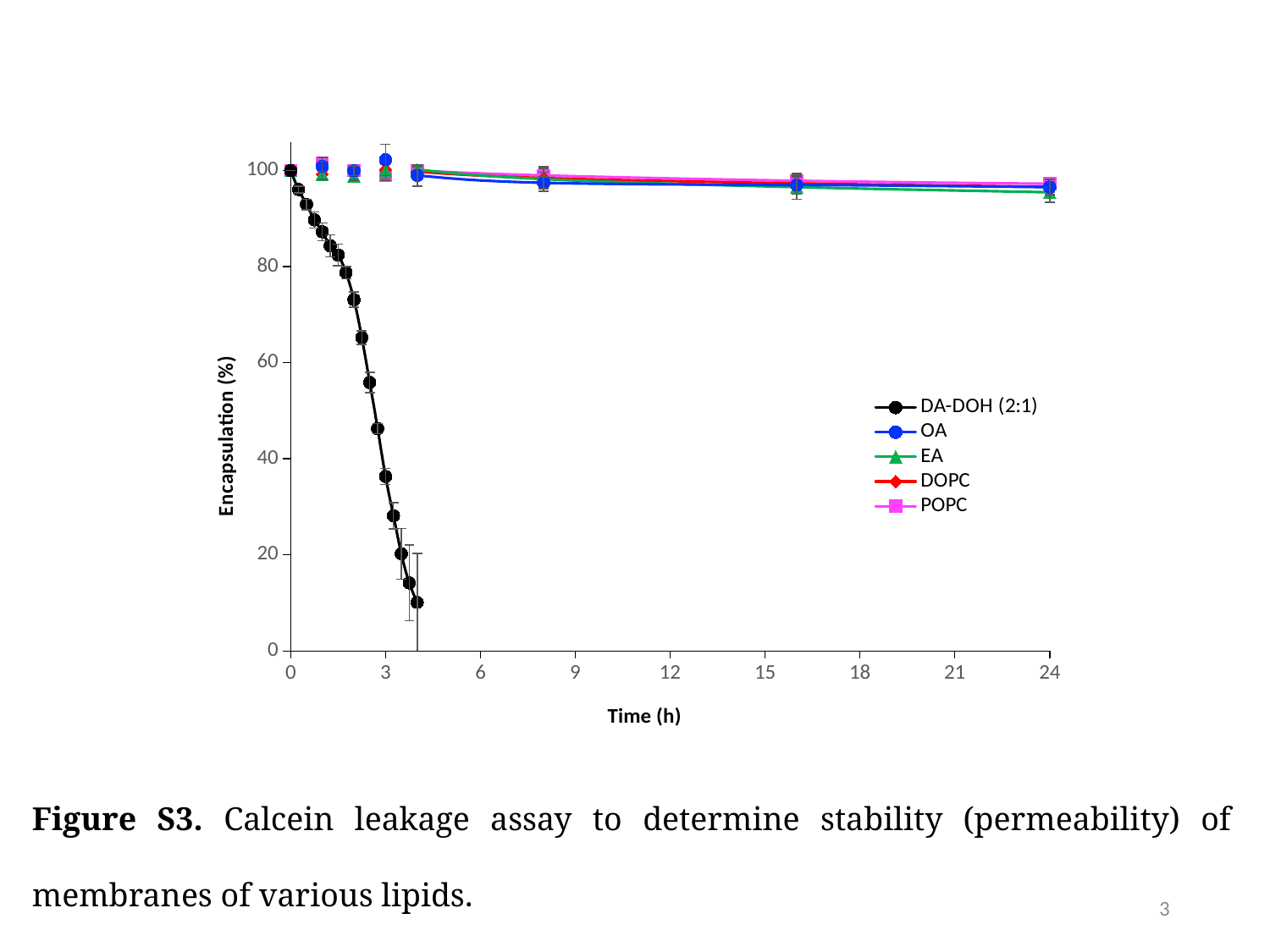

### Chart
| Category | | | | | |
|---|---|---|---|---|---|Figure S3. Calcein leakage assay to determine stability (permeability) of membranes of various lipids.
3

## Slide 4
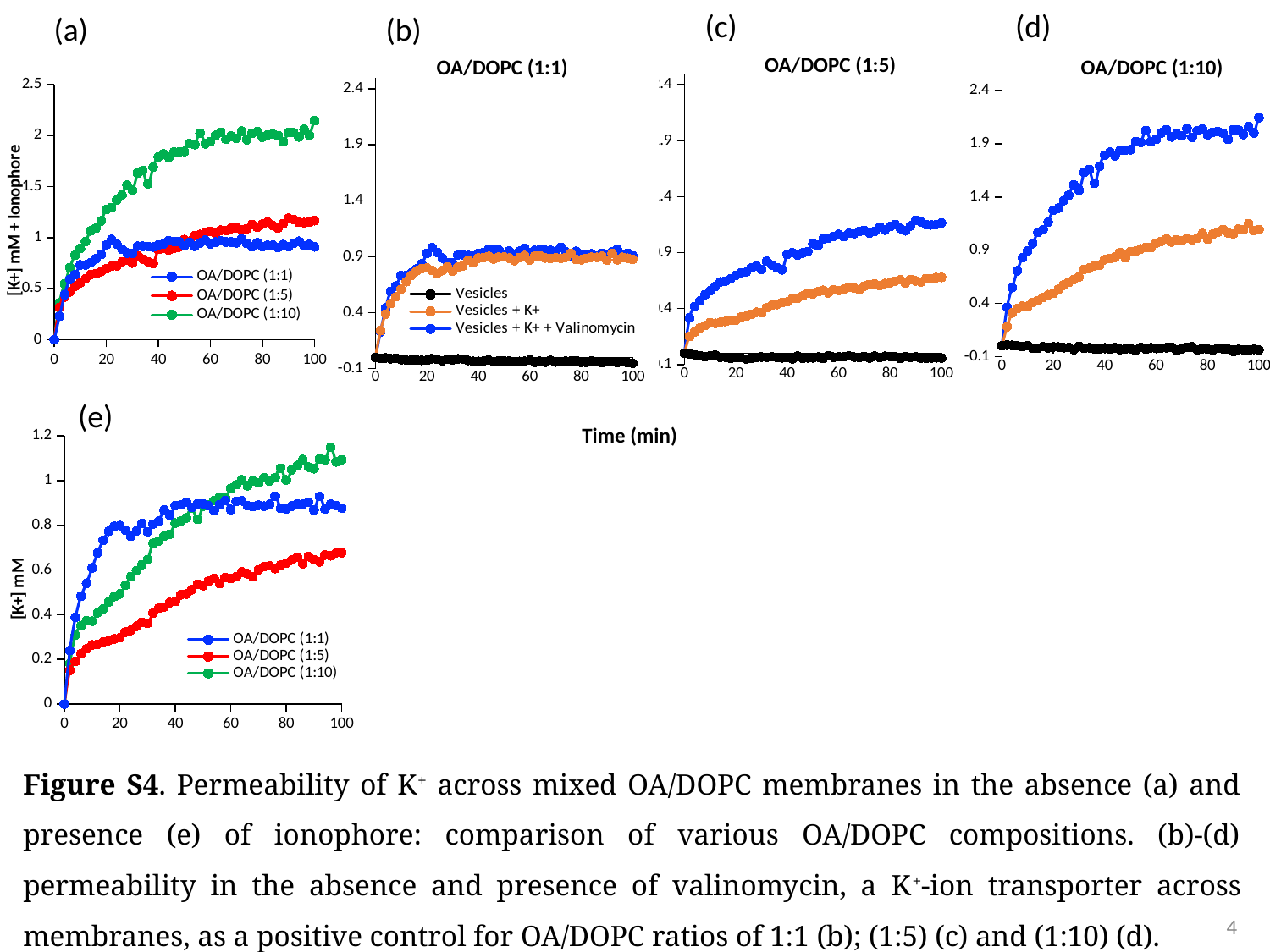

(c)
(d)
(a)
(b)
OA/DOPC (1:5)
OA/DOPC (1:1)
OA/DOPC (1:10)
### Chart
| Category | | | |
|---|---|---|---|
### Chart
| Category | | | |
|---|---|---|---|
### Chart
| Category | | | |
|---|---|---|---|
### Chart
| Category | | | |
|---|---|---|---|(e)
Time (min)
### Chart
| Category | | | |
|---|---|---|---|Figure S4. Permeability of K+ across mixed OA/DOPC membranes in the absence (a) and presence (e) of ionophore: comparison of various OA/DOPC compositions. (b)-(d) permeability in the absence and presence of valinomycin, a K+-ion transporter across membranes, as a positive control for OA/DOPC ratios of 1:1 (b); (1:5) (c) and (1:10) (d).
4

## Slide 5
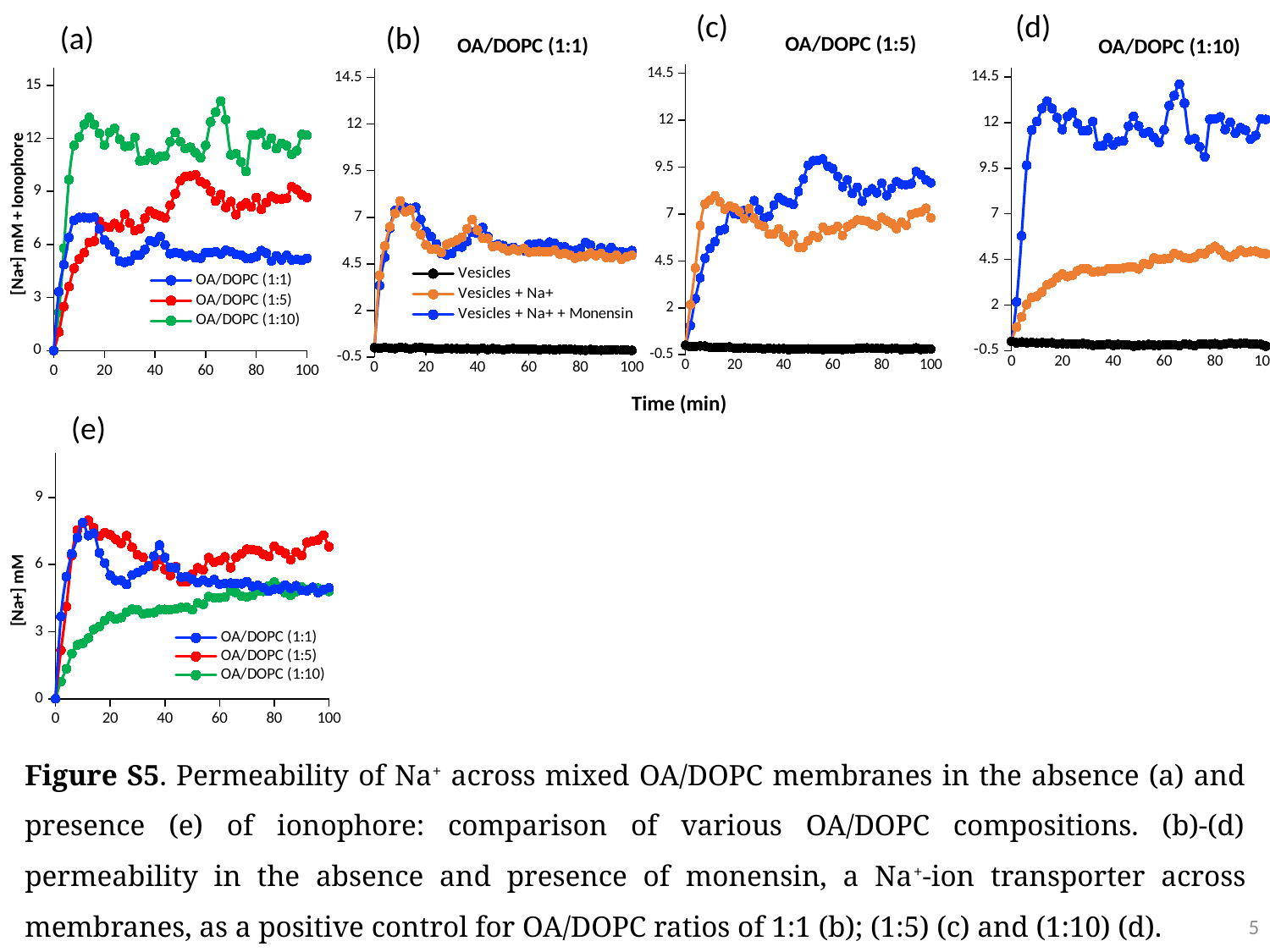

(c)
(d)
(a)
(b)
OA/DOPC (1:5)
OA/DOPC (1:1)
OA/DOPC (1:10)
### Chart
| Category | | | |
|---|---|---|---|
### Chart
| Category | | | |
|---|---|---|---|
### Chart
| Category | | | |
|---|---|---|---|
### Chart
| Category | | | |
|---|---|---|---|Time (min)
(e)
### Chart
| Category | | | |
|---|---|---|---|Figure S5. Permeability of Na+ across mixed OA/DOPC membranes in the absence (a) and presence (e) of ionophore: comparison of various OA/DOPC compositions. (b)-(d) permeability in the absence and presence of monensin, a Na+-ion transporter across membranes, as a positive control for OA/DOPC ratios of 1:1 (b); (1:5) (c) and (1:10) (d).
5

## Slide 6
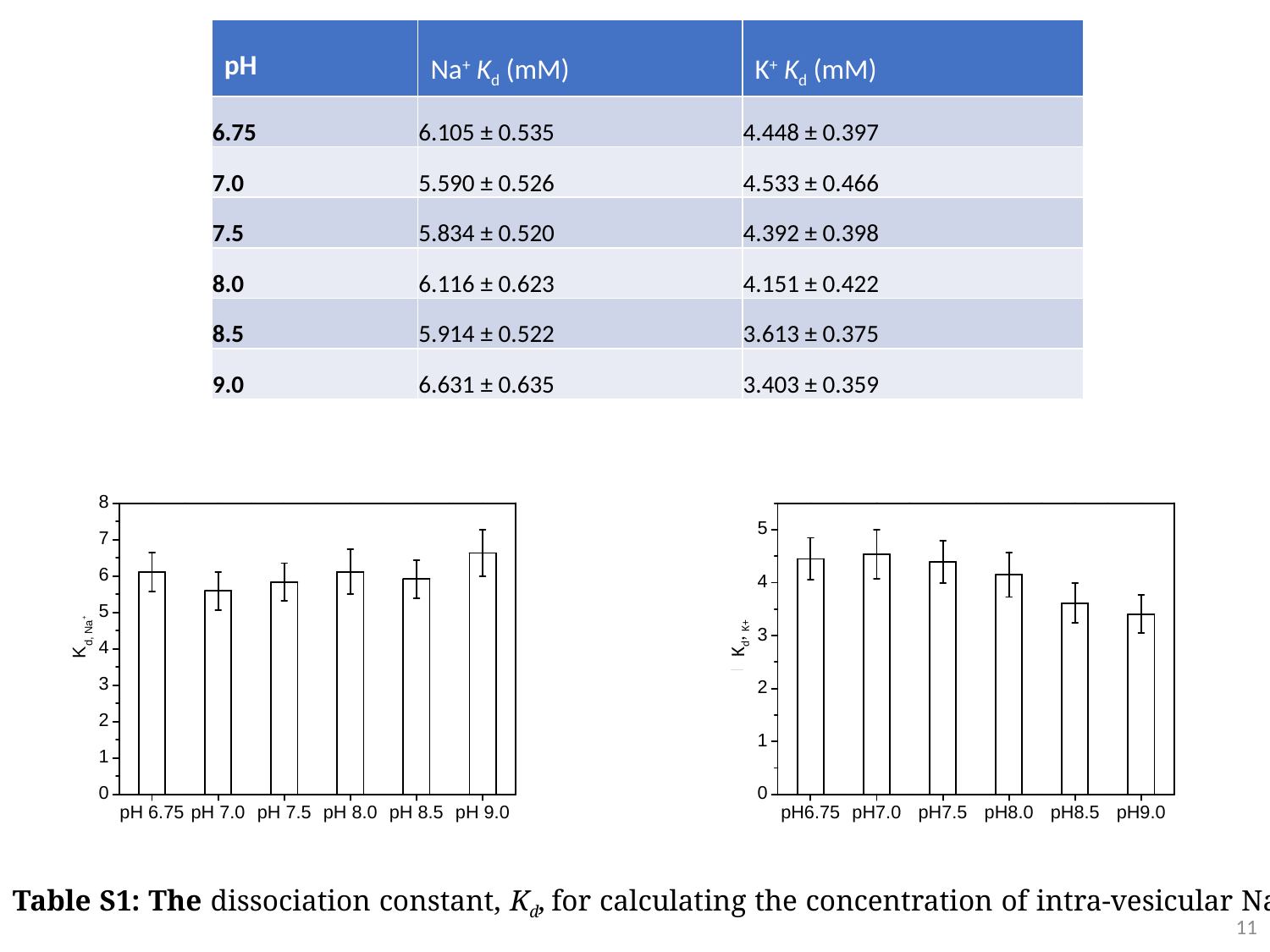

| pH | Na+ Kd (mM) | K+ Kd (mM) |
| --- | --- | --- |
| 6.75 | 6.105 ± 0.535 | 4.448 ± 0.397 |
| 7.0 | 5.590 ± 0.526 | 4.533 ± 0.466 |
| 7.5 | 5.834 ± 0.520 | 4.392 ± 0.398 |
| 8.0 | 6.116 ± 0.623 | 4.151 ± 0.422 |
| 8.5 | 5.914 ± 0.522 | 3.613 ± 0.375 |
| 9.0 | 6.631 ± 0.635 | 3.403 ± 0.359 |
Kd, K+
Table S1: The dissociation constant, Kd, for calculating the concentration of intra-vesicular Na+ or K+.
11

## Slide 7
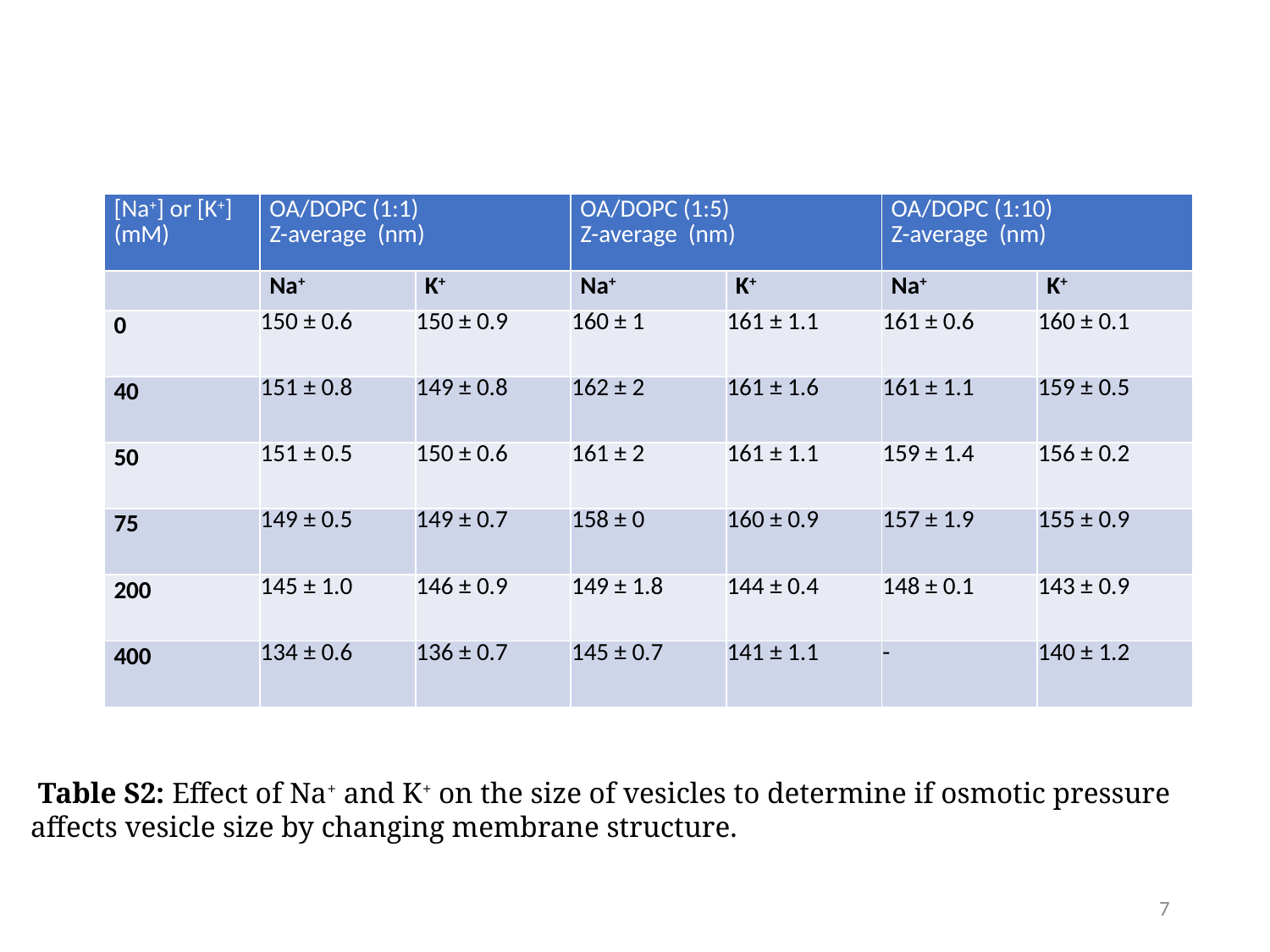

| [Na+] or [K+] (mM) | OA/DOPC (1:1) Z-average (nm) | | OA/DOPC (1:5) Z-average (nm) | | OA/DOPC (1:10) Z-average (nm) | |
| --- | --- | --- | --- | --- | --- | --- |
| | Na+ | K+ | Na+ | K+ | Na+ | K+ |
| 0 | 150 ± 0.6 | 150 ± 0.9 | 160 ± 1 | 161 ± 1.1 | 161 ± 0.6 | 160 ± 0.1 |
| 40 | 151 ± 0.8 | 149 ± 0.8 | 162 ± 2 | 161 ± 1.6 | 161 ± 1.1 | 159 ± 0.5 |
| 50 | 151 ± 0.5 | 150 ± 0.6 | 161 ± 2 | 161 ± 1.1 | 159 ± 1.4 | 156 ± 0.2 |
| 75 | 149 ± 0.5 | 149 ± 0.7 | 158 ± 0 | 160 ± 0.9 | 157 ± 1.9 | 155 ± 0.9 |
| 200 | 145 ± 1.0 | 146 ± 0.9 | 149 ± 1.8 | 144 ± 0.4 | 148 ± 0.1 | 143 ± 0.9 |
| 400 | 134 ± 0.6 | 136 ± 0.7 | 145 ± 0.7 | 141 ± 1.1 | - | 140 ± 1.2 |
 Table S2: Effect of Na+ and K+ on the size of vesicles to determine if osmotic pressure affects vesicle size by changing membrane structure.
7
